# 1-MHz linewidth VCSEL enabled by monolithically integrated passive cavity for high-stability chip-scale atomic clocks

**DOI:** 10.1038/s41377-026-02192-x

**Published:** 2026-01-29

**Authors:** Zhiting Tang, Chuanlin Li, Xuhao Zhang, Wuyang Ren, Kai Shen, Chuang Li, Qingsong Bai, Jin Li, Aobo Ren, Hao Wang, Xiaorong Luo, Hongxing Xu, Jiang Wu

**Affiliations:** 1 Institute of Fundamental and Frontier Sciences, University of Electronic Science and Technology of China, Chengdu, 611731 China; 2 Chengdu Spaceon Electronics Corporation Ltd., Chengdu, 610036 China; 3 School of Instrumentation and Optoelectronic Engineering, Beihang University, 100191 Beijing, China; 4 Division of Electrical Engineering, Department of Engineering, University of Cambridge, Cambridge, CB3 0FA UK; 5 College of Microelectronics, Chengdu University of Information Technology, Chengdu, China; 6 State Key Laboratory of Electronic Thin Films and Integrated Devices, University of Electronic Science and Technology of China, Chengdu, 611731 China; 7 Mozi Laboratory, Zhengzhou, 450001 China

**Keywords:** Semiconductor lasers, Photonic devices

## Abstract

Narrow-linewidth vertical-cavity surface-emitting lasers (VCSELs) are key enablers for chip-scale atomic clocks and quantum sensors, yet conventional designs suffer from short cavity lengths and excess spontaneous emission, resulting in broad linewidths and degraded frequency stability. Here, we demonstrate a monolithically integrated VCSEL operating at the cesium D_1_ line (894.6 nm) that achieves intrinsic linewidth compression to ~1 MHz, without requiring external optical feedback. This performance is enabled by embedding a passive cavity adjacent to the active region, which spatially redistributes the optical field into a low-loss region, extending photon lifetime while suppressing higher-order transverse and longitudinal modes. The resulting device exhibits robust single-mode operation over a wide current and temperature range, with side-mode suppression ratio (SMSR) > 35 dB, orthogonal polarization suppression ratio (OPSR) > 25 dB and a beam divergence of ~7°. Integrated into a Cesium vapor-cell atomic clock, the VCSEL supports a frequency stability of 1.89 × 10^–12^ τ^-1/2^. These results position this VCSEL architecture as a compact, scalable solution for next-generation quantum-enabled frequency references and sensing platforms.

## Abstract (web summary)

This work demonstrates a monolithically integrated VCSEL with an embedded passive cavity, achieving an ultranarrow ~1 MHz linewidth and enabling high-stability chip-scale atomic clocks.

## Graphical abstract

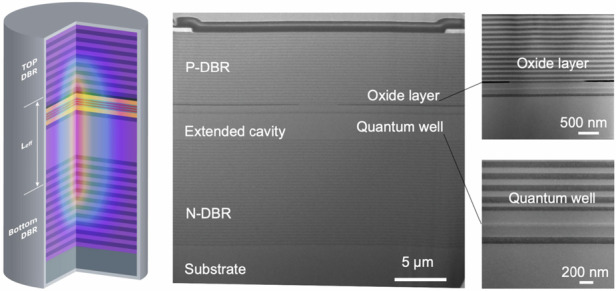

## Introduction

Next-generation quantum devices, including chip-scale atomic clocks, optical gyroscopes, and magnetometers, are being deployed in positioning, navigation, and timing (PNT) systems, aerospace, and precision metrology^1–3^. These advanced quantum sensors require ultra-narrow linewidths, stable single-mode emission, and consistent optical output across varying environmental conditions, as well as highly integrated, miniaturized light sources suitable for chip-scale integration. Combining precise spectral performance with a compact form factor is thus essential for realizing practical and scalable quantum sensing systems. Vertical-cavity surface-emitting lasers (VCSELs) are emerging as essential light sources for such precision applications. Owing to their low threshold currents^4,5^, low beam divergence^6,7^, and planar structures amenable to large-scale integration^8–11^, VCSELs are well-suited for compact and efficient coherent light generation. However, their inherently short cavity lengths and thus high round-trip losses limit photon lifetime, resulting in emission linewidths typically exceeding 100 MHz^12^, well above the ideal threshold required for quantum applications. Laser phase noise, which manifests as a broadened linewidth, can easily converts to amplitude noise in atomic system^13,14^, causing absorption signal fluctuations and degrading the precision and stability of quantum sensors^15,16^. Therefore, developing narrow-linewidth VCSELs with enhanced spectral purity and stability has become a priority for enabling next-generation integrated quantum sensing platforms.

To address these limitations, various linewidth-narrowing strategies have been explored, including self-injection locking to high-*Q* resonators^17^, external cavity configurations^18^, and active stabilization techniques such as Pound–Drever–Hall locking^19^. While these methods can compress linewidths to sub-megahertz or even kilohertz levels, they fundamentally rely on external resonators, such as microrings^20,21^, liquid-crystal films^22^, or bulk optical cavities^23^, which increase system complexity, alignment sensitivity, physical footprint, and fabrication costs. Their dependence on external components thus limits their suitability for compact, robust, chip-scale quantum sensors. An alternative solution involves monolithically integrating internal cavities to extend the effective cavity length of a VCSEL^24^. This approach leverages cavity design to mitigated optical losses and enhance photon lifetime within a fully integrated structure, enabling linewidth narrowing to the few-megahertz range while preserving VCSELs’ inherent compactness and integrability. However, such architectures introduce critical trade-offs: as the cavity length increases, the free spectral range (FSR) shrinks^25^, making it increasingly difficult to maintain single-longitudinal-mode operation—a limitation commonly known as the “FSR wall.” Simultaneously, transverse mode behavior is influenced by carrier-induced refractive index changes, diffraction, thermal gradients, and oxide-aperture index guiding^26,27^. These factors exert comparable influence and must be managed alongside longitudinal mode control within a unified cavity design.

Therefore, precise cavity engineering is essential to balance linewidth compression with the simultaneous control of longitudinal and transverse modes. Achieving high *Q*-factors within a monolithic architecture, where the optical mode volume is inherently limited, remains technically challenging. Moreover, narrower linewidths heighten the device’s susceptibility to mode hopping, thermal fluctuations, and carrier-induced noise. Ensuring low noise and stable single-mode operation over wide temperature and power ranges thus requires advanced structural design and fabrication control.

Here, we demonstrate a passive-cavity embedded VCSEL that achieves narrow linewidth and stable single-mode operation over a broad current and temperature range. The architecture enhances the effective cavity length by spatially redistributing the optical mode into a low-loss passive cavity, effectively reducing the spontaneous emission. This structure is also found to suppress parasitic transverse modes. Without the need for external optical feedback, the device achieves a low frequency noise of ~2.3 × 10⁵ Hz²/Hz and an intrinsic linewidth of approximately 1 MHz. Operating at the cesium (Cs) D_1_ transition wavelength (894.6 nm), the device exhibits excellent frequency stability, making it a promising candidate for quantum sensing applications.

## Results

### Concept and implementation

Semiconductor lasers inherently exhibit frequency fluctuations due to spontaneous emission, which introduces random phase variations and contributes to linewidth broadening^14^, further exacerbated by cavity losses, etc. In our proposed design, we integrate a passive cavity section into the bottom distributed Bragg reflectors (DBRs) (rather than top DBRs) to minimize free carrier absorption in the passive cavity, thereby providing phase-fixed feedback. The design promotes a longer effective cavity by redistributing the optical mode toward the low-loss passive cavity, thereby minimizing the impact of spontaneous emission and substantially compressing the laser linewidth.

**Fig. 1:**
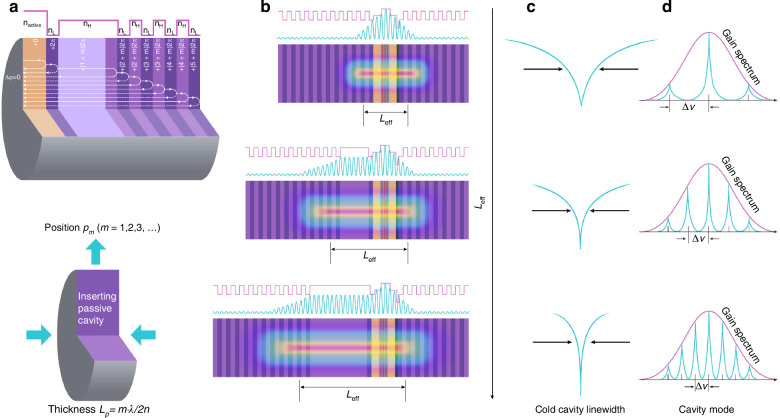
Operating principle and trade-offs associated with optical mode volume in VCSELs. **a** Schematic illustration of a passive-cavity-embedded VCSEL. To achieve enhanced reflectivity through constructive interference, the relative phase difference between two neighboring reflected beams must be an integer multiple of 2π. **b** Standing-wave electric field distribution as a function of the passive cavity length *L*_*p*_. **c** Increasing the *L*_*p*_ results in a longer effective cavity length *L*_eff_, leading to significant compression of the cold-cavity linewidth. **d** As the cavity length increases, the FSR decreases, making it harder to maintain single-longitudinal-mode operation

Two key design parameters are considered (Fig. 1a): the position of the embedded passive section (*P*_*m*_, where *m* = 1, 2, 3, …) within the DBRs and the length of the passive cavity (*L*_*p*_). The *P*_*m*_ directly influences the penetration depth of the standing wave from the main cavity, defined as the point where the electric field intensity drops to 1/*e* of its maximum value in the active region^28^. Light interacting with a multilayer film undergoes two key processes: phase accumulation during propagation and discrete phase shift upon reflection. Constructive interference requires a 2π phase difference between successive beams. The *L*_*p*_ must strictly satisfy the transmission phase condition (2π·*m*), and it determines the photon density feedback into the main cavity. These two factors, in turn, effectively reduces the coupling between spontaneous radiation and stimulated emission, ultimately influencing the effective cavity length (*L*_eff_) on the mirror side and contributing to linewidth narrowing during laser oscillation.

A primary design rule dictates that the *L*_eff_ should be as long as possible. As illustrated in Fig. 1b, we observe that increasing the *L*_*p*_ leads to an increase in the *L*_eff_, resulting in a notable compression of the cold cavity linewidth (Fig. 1c). However, this extension also reduces the FSR (Fig. 1d), which in turn increases competition between multiple longitudinal modes within the gain bandwidth of the active region. Therefore, two conditions should be met: (1) the standing wave electric field in the passive cavity must remain above the DBRs’ penetration depth threshold to ensure sufficient field strength for photons; and (2) competition between multiple longitudinal modes must be avoided to maintain stable single-mode operation.

**Fig. 2:**
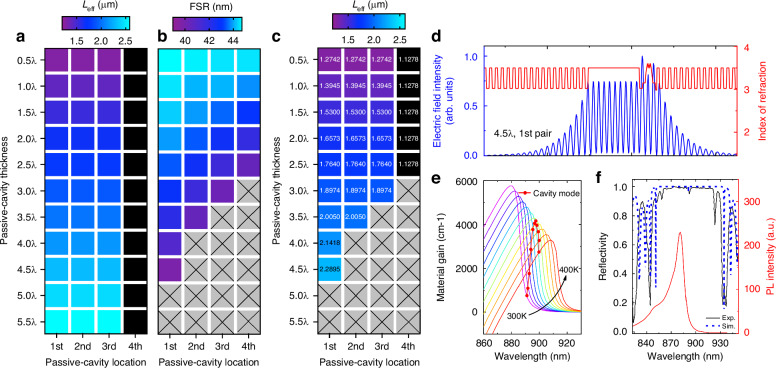
Cavity design and structural implementation. **a** Heatmap of *L*_eff_ as a function of *L*_*p*_ and mode positioning *P*_*m*_ within the bottom DBR pairs. **b** Heatmap of FSR derived from the same parameter space, overlaid with the gain spectrum. Cavity modes become densely packed for different passive-cavity location (e.g., *L*_*p*_ > 5λ). **c** Optimal cavity configuration identified at *L*_*p*_ = 4.5λ, *P*_*m*=1_, yielding an *L*_eff_ of 2.29 μm while balancing linewidth narrowing and single-mode stability. **d** Refractive index profile and normalized electric field distribution of the optimized structure. The oxide aperture is aligned with a field node to minimize absorption, while the quantum wells are placed at antinodes to maximize modal gain. **e** Simulated gain spectra of the active region and cavity mode as a function of temperature. A designed detuning of –12.1 nm ensures spectral overlap at elevated operating temperatures by compensating for gain redshift. **f** Measured (solid line) and calculated (dashed line) reflection and PL spectra of the as-grown epi-wafer, confirming cavity-mode detuning and strong optical gain at the target lasing wavelength

Building on the design framework outlined above, we examined all possible cavity structures and calculated their *L*_eff_ for various *L*_*p*_ and different *P*_*m*_. The *L*_*p*_ is set as $m$⋅${\rm{\lambda }}/2n$ (where *m* = 1, 2, 3, …), providing a round-trip phase of 2π·*m*, with *n* being the refractive index of the passive cavity. This phase relationship ensures constructive multi-beam interference. The heat map of the extracted *L*_eff_ (*L*_*p*_, *P*_*m*_), based on the corresponding optical field distribution (Fig. S1), is shown in Fig. 2a. We observed an increase in *L*_eff_ and *Q*-factors as *L*_*p*_ increased (Fig. S2 and S3). Additionally, we noted that *L*_eff_ remained stable for *P*_*m*_ between the first and third pairs of bottom DBRs. In contrast, for *P*_*m*_ beyond the fourth DBR pair, *L*_eff_ did not show any significant extension, indicating that the electric field of the standing wave decayed below the penetration depth threshold, losing its ability to provide sufficient optical confinement for photons.

While increasing *L*_eff_ theoretically narrows the spectral linewidth due to an extended photon lifetime, it also impacts longitudinal mode dynamics. The reduction in FSR limits how much the linewidth can be suppressed. To evaluate this effect, we generated a heat map of FSR as a function of *L*_*p*_ and *P*_*m*_ aligning the calculated cavity modes with the net gain spectrum (Fig. 2b and Fig. S4). The cavity mode dip appears near 891.6 nm, while the PL spectrum peaks at 882.1 nm with a full width at half maximum (FWHM) of 39.5 nm, *e.g*., broader than the mode spacing for a cavity length of 5λ or greater at *P*_n=1_. Thus, we clearly observe the “FSR wall” across various *L*_*p*_ and *P*_*m*_ values. Based on these comprehensive structural analyses and boundary condition constraints, we found that a 4.5λ-long *L*_*p*_ at *P*_*m*=1_ provides the optimal configuration with a *L*_eff_ of 2.29 μm. (Fig. 2c). This ensures sufficient spectral overlap for mode selection while highlighting the importance of carefully balancing cavity length and mode spacing for stable single-mode operation. Furthermore, we calculated the spontaneous emission rates across various cavity configurations, confirming that the introduction of the passive cavity structure significantly contributes to reduction of optical losses and quantum limited white noise (Fig. S5).

Figure 2d shows the schematic of the VCSEL structure screened based on boundary conditions, along with the corresponding refractive index and electric field distribution. An oxide layer, located at the standing wave node, serves as both an electrical aperture and an optical confinement layer while minimizing absorption. The multiple quantum wells (MQWs) are positioned at the antinode of the standing wave to maximize optical gain. VCSELs designed for miniaturized atomic clock microsystems must operate reliably at elevated temperatures. However, heating of the active region can cause the emission wavelength to drift away from the target. Although the cavity mode shifts relatively slowly (~0.05 nm·K⁻¹), the faster drift of the gain peak (~0.3 nm·K⁻¹) can be compensated by engineering the gain–cavity detuning. As illustrated in Fig. 2e, a detuning of –12.1 nm is selected to maintain stable emission at high temperatures (e.g., 360 K).

Following the design specifications, the epitaxial layers were grown on an Si-doped GaAs substrate using metal-organic chemical vapor deposition (MOCVD) (see Method). Figure 2f shows the measured reflection and PL spectrum of the as-grown epi-wafer. The observed reflection bandwidth and Fabry–Perot (FP) dip aligns well with the simulated results, supporting strong mode gain and precise lasing.

### Device optoelectronic performance

**Fig. 3:**
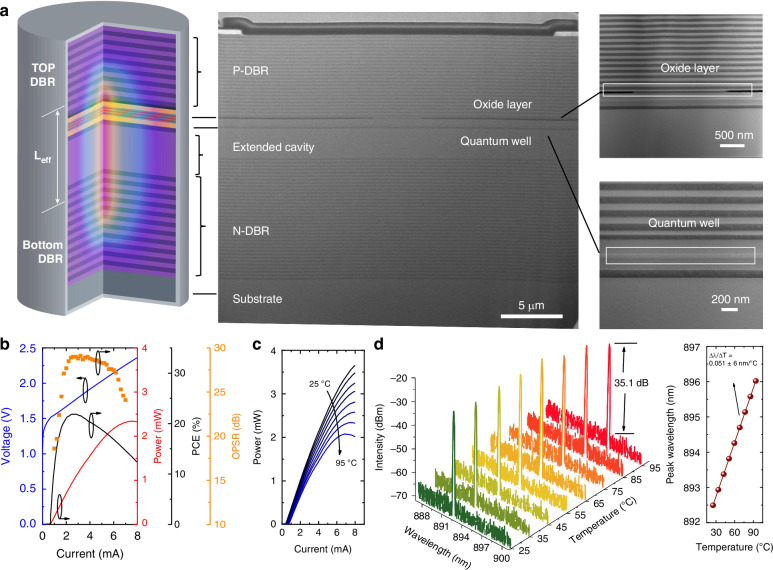
Device architecture and optoelectronic performance. **a** Schematic illustration and corresponding cross-sectional SEM image of the device, comprising *n*-type DBRs, a passive cavity, multiple quantum wells, an oxide layer, and *p*-type DBRs. Scale bar: 5 μm, 500 nm, 200 nm. **b** L-I-V-OPSR characteristics of the VCSEL measured at 85 °C. **c** L-I curves of the device measured at various temperatures ranging from 25 °C to 95 °C. **d** Emission spectra measured at different temperatures (25 °C to 95 °C) under a fixed injection current of 1.4 mA. The inset shows the wavelength shift (∆λ/∆T), exhibiting a linear redshift of 0.05 nm/°C with increasing ambient temperature

VCSELs were fabricated using a standard III-V wafer processing flow (Fig. S6). Figure 3a shows the DBRs, active region, passive cavity, and the etched mesa structure with a 3.5 μm oxide aperture. The light–current–voltage (L–I–V) characteristics, power conversion efficiency (PCE), and orthogonal polarization suppression ratio (OPSR) of a proposed device were measured under continuous-wave (CW) operation, as shown in Fig. 3b. The device demonstrates a low threshold current of 0.76 mA, a maximum output power of 2.2 mW, a peak PCE of 20%, and an OPSR exceeding 25 dB at 85 °C. The L–I characteristics (Fig. 3c) and PCE curves (Fig. S7) show only a slight thermal roll-off in output power and a modest decline in PCE over a case temperature range from 25 to 95 °C, attributed to thermally induced carrier leakage. The threshold current remains well below 1.0 mA and increases only slightly to 0.84 mA at 95 °C, due to the need for higher carrier densities to compensate for reduced material gain at elevated temperatures. Figure 3d displays the emission spectra at various temperatures under a constant injection current of 1.4 mA. The device maintains stable single-mode operation with a side-mode suppression ratio (SMSR) of 35.1 dB, even at temperatures up to 95 °C. The target emission wavelength of 894.6 nm, aligned with the Cs D_1_ transition line, is achieved at 75 °C with an injection current of 1.4 mA, accompanied by a high SMSR of 40 dB and an OPSR exceeding 29 dB (see polarization-resolved spectra in Fig. S8). A typical linear redshift in peak wavelength with increasing ambient temperature, caused by temperature-induced changes in the material’s refractive index, yields a tuning coefficient of ∆λ/∆T = 0.05 nm/°C (inset of Fig. 3d).

**Fig. 4:**
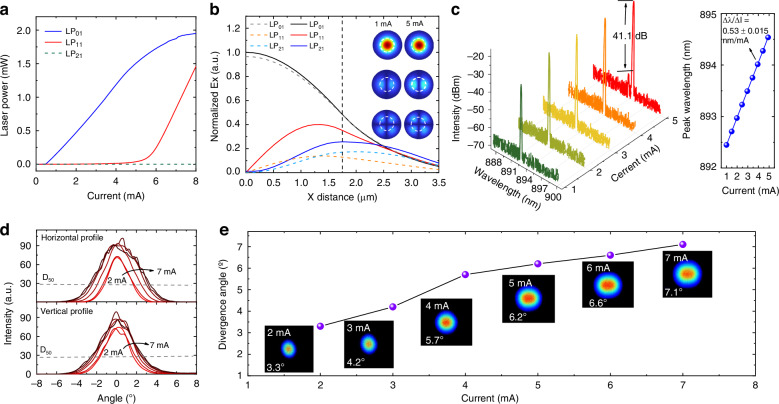
Fundamental transverse modes behaviors. **a** Calculated L‐I curves of LP_01_, LP_11_, and LP_21_ transverse modes. **b** Calculated radial intensity distributions of the LP₀₁, LP₁₁, and LP₂₁ transverse modes at injection currents of 1 mA (dashed curves) and 5 mA (solid curves). The oxide aperture boundary is marked by the vertical dashed line. The inset presents corresponding 2D mode profiles, illustrating the distinct field confinement characteristics of each mode. **c** Emission spectra at different injection currents (1–6 mA) at room temperature (25 °C). The inset displays the peak wavelength shift (Δλ/ΔI), showing a linear tuning coefficient of 0.53 nm/mA. **d** Divergence angle of the output beam measured in two orthogonal directions. The dashed lines represent the D_50_ divergence profile. **e** Far-field intensity profiles at 85 °C under various drive currents (2–7 mA), demonstrating consistently low beam divergence below ~7.0°

We next examine the behavior of the fundamental and higher-order linearly polarized (LP) transverse modes in the device. Figure 4a presents the calculated L–I curves for different transverse modes. The threshold current for the LP₀₁ mode and the LP₁₁ mode occurring at approximately 0.63 mA and 5.25 mA. No higher-order modes appear even at elevated current levels, indicating stable single-mode operation. Figure 4b presents both the 1D and 2D intensity distributions of the first three transverse modes (LP₀₁, LP₁₁, and LP₂₁) along the radial direction of the VCSEL aperture. The LP₀₁ mode is initially excited and remains dominant, while the LP₁₁ mode emerges at a drive current of 5 mA. This large threshold separation ensures a broad and stable output power range for fundamental-mode operation, as further confirmed by the current-dependent lasing spectra (Fig. 4c). Stable LP₀₁-mode emission is maintained from threshold up to 5 mA, delivering a maximum output power of 2.6 mW with a side-mode suppression ratio (SMSR) consistently exceeding 40 dB. Additionally, the peak wavelength exhibits a linear redshift with increasing injection current, characterized by ∆λ/∆I = 0.53 nm/mA. This fine-tuning capability offers excellent linearity and tunability, making it highly advantageous for system integration.

This enhanced single-mode behavior is attributed to the embedded passive cavity, which increases diffraction losses for higher-order transverse modes. In oxide-confined VCSELs, transverse optical confinement is achieved by selectively oxidizing a high-aluminum-content Al₀.₉₈Ga₀.₀₂As layer to form an Al₂O₃ cladding with a significantly lower refractive index. This creates a cylindrical waveguide structure, where the index contrast between the unoxidized core and oxidized cladding, quantified by the effective refractive index difference ∆*n*_eff_, governs the number and order of supported LP modes (see Method). We calculated ∆*n*_eff_ as a function of the *P*_*m*_ and *L*_*p*_ embedded in the DBRs (Fig. S9). The results indicate that our proposed structure yields the smallest ∆*n*_eff_ among all configurations considered, providing optimal suppression of higher-order modes.

A reduced ∆*n*_eff_ also results in a smaller beam divergence and improved beam quality. Figure 4d presents the far-field patterns under varying injection currents. From 2 mA to 5 mA, the beam exhibits a clean Gaussian profile, confirming single transverse-mode operation. Above 5 mA, the far-field patterns begin to show contributions from higher-order modes. The beam divergence angle, defined as the angle at which the D₅₀ beam width expands linearly with distance, was extracted from these measurements. The D₅₀ beam width refers to the diameter of a circle centered at the beam centroid that contains 50% of the total far-field power. As shown in Fig. 4e, the divergence angle remains as low as 7.0° at the maximum single-mode current of 5 mA, significantly smaller than the typical values, e.g., ~15°, of conventional VCSELs. According to the equation in (9), the effective refractive index difference was calculated to be only 0.0035 which is smaller than the conventional VCSEL of 0.006 ~ 0.01 [27]. The suppression of higher-order modes, facilitated by the small effective index difference, enhances modal purity and consequently reduces beam divergence.

### Laser noise and frequency stability

**Fig. 5:**
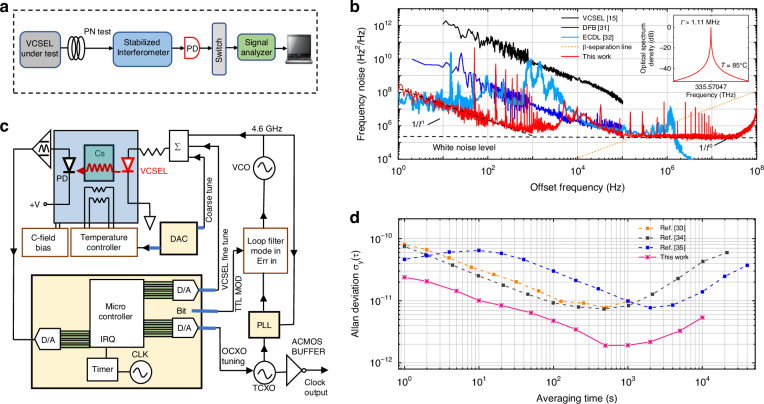
Frequency noise characteristics and atomic clock integration. **a** Experimental setup for frequency noise measurement based on a homodyne detection scheme. The laser output is mixed with a delayed replica to extract the frequency noise PSD. **b** Single-side frequency noise PSD of the proposed VCSEL, compared with state-of-the-art free-running laser sources. The inset shows the corresponding Lorentzian linewidth derived from the PSD, with the dashed line indicating the β-separation line that delineates coherence-dominant noise. **c** Schematic of the cesium atomic clock system incorporating the VCSEL. Key components include the VCSEL-driven Cs vapor-cell physics package, microwave generation and feedback control loop, and a digital microcontroller. **d** Measured Allan deviation (σᵧ(τ)) of the VCSEL-based Cs clock, showing stable frequency performance up to a tracking duration of 10,000 s. For comparison, results from three reference systems are also shown

The frequency stability of a laser can be quantitatively described by its frequency noise power spectral density (PSD), which reflects the temporal fluctuations in its emission frequency. By integrating the frequency noise PSD over all Fourier frequencies, one can derive the laser’s spectral lineshape, as governed by Eq. (26)^29^ (see Method). To experimentally evaluate the frequency noise characteristics of our VCSEL, a fiber-based Mach–Zehnder interferometer was used as a frequency discriminator (Fig. 5a). The system incorporates a temperature-controlled platform and a low-noise fiber coupling setup, ensuring minimal thermal and mechanical interference during measurement. The output is processed by a high-resolution frequency noise analyzer.

Figure 5b shows the measured PSD of the VCSEL operating at 1.4 mA. The spectrum exhibits two distinct regions: at low offset frequencies, 1/*f*-type flicker noise dominates, primarily caused by technical noise sources such as mechanical vibrations, current fluctuations, and thermal drift. At higher frequencies, these effects diminish, and the spectrum transitions to a flat white noise floor attributed to quantum noise from spontaneous emission and carrier fluctuations. This white noise floor directly determines the Lorentzian linewidth, which remains nearly constant (~2.3 × 10⁵ Hz²/Hz) over a wide temperature range (25–95 °C), corresponding to a linewidth of approximately 1.0 MHz (cross-validated by heterodyne beat-note measurement, Fig. S10).

For benchmarking, the frequency noise characteristics of our VCSEL were compared with those of two state-of-the-art free-running semiconductor lasers over the 1–100 MHz offset range. The linewidths were estimated using the β-separation line method^30^, defined by S_*ν*_ (*f*)=8·ln(2)*f*/ π^2^. By integrating the frequency noise PSD up to the intersection with this line, the full width at half maximum (FWHM) linewidth can be approximated for a given integration time. Using this approach, our VCSEL achieves linewidths of 1.11 MHz at 0.1 ms, 1.21 MHz at 1 ms, 1.28 MHz at 10 ms, and 1.31 MHz at 100 ms, all of which are significantly narrower or at least comparable to those of advanced free-running laser sources, including a conventional VCSEL (20–25 MHz), a distributed feedback (DFB) laser (<1 MHz), and an external cavity diode laser (ECDL, ~1 MHz)^15,31,32^. These results demonstrate that the embedded passive cavity effectively suppresses spontaneous emission noise, resulting in reduced frequency noise and enhanced coherence.

We next evaluate the stability of a clock implemented within a vapor-phase CW cesium atomic clock testbed. Figure 5c illustrates the functional blocks of a VCSEL-based coherent population trapping (CPT) atomic clock. The proposed VCSEL is temperature-stabilized and frequency-locked to the Cs D₁ transition at 894.6 nm using linear absorption spectroscopy. A voltage-controlled crystal oscillator (VCXO) provides reference input to a microwave synthesizer, which generates a 9.2 GHz signal to drive the microwave cavity and excite the ground-state hyperfine (0–0) transition in cesium vapor. The microwave power is adjusted by tuning the synthesizer output level.

The frequency stability of the clock was rigorously evaluated through Allan deviation σ_y_ (τ) measurements, as shown in Fig. 5d. At short averaging times, the Allan deviation follows a clear τ^−1/2^ dependence, characteristic of white frequency noise, with σ_y_ (1 s) ≈ 2 × 10⁻¹¹. As the averaging time increases, the stability improves until it reaches a flicker noise floor at around 300 s, with a minimum Allan deviation of σ_y_ (τ) = 1.89 × 10^⁻¹²^. This level marks the intrinsic short-term stability limit of the current system configuration. This high stability is primarily attributed to the low frequency noise of the passive-cavity embedded VCSEL, which is actively stabilized using a lock-in detection scheme. Notably, at the flicker floor, the VCSEL is not the dominant source of noise, underscoring its suitability for frequency-sensitive applications. Beyond 300 s, the Allan deviation begins to rise due to the onset of low-frequency noise processes such as flicker frequency noise or random walk.

To evaluate the performance of our system, we benchmarked it against three representative VCSEL-based Cs clocks over time^33–35^ (Fig. 5d). The three reference systems show minimum Allan deviations of 7.36 × 10⁻¹² τ^−1/2^, 7.71 × 10⁻¹² τ^−1/2^, 7.78 × 10⁻¹² τ^−1/2^, respectively. Owing to the reduced frequency noise and narrow spectral linewidth of our VCSEL, the resulting optical clock exhibits significantly improved frequency stability over a tracking duration of up to 10,000 s. This performance demonstrates that our VCSEL achieves superior frequency stability compared to the state-of-the-art VCSEL-based atomic clocks, highlighting its potential to redefine the capabilities of chip-scale frequency standards.

## Discussions

In summary, we have demonstrated a monolithic, passive-cavity embedded VCSEL operating at 894.6 nm, achieving a 1 MHz linewidth alongside excellent modal purity and beam quality. The device features a carefully engineered cavity architecture that maximizes photon lifetime while suppressing higher-order transverse and longitudinal modes. This enables robust single-mode operation without external feedback or additional mode-selective components, streamlining fabrication and enhancing stability. The VCSEL delivers strong performance metrics, including a SMSR > 35 dB, OPSR > 25 dB, low beam divergence of ~7.0°, and high thermal robustness, meeting the stringent demands of integrated quantum systems.

When integrated into a Cs vapor-cell CPT clock, the device supports an excellent frequency stability of 1.89 × 10^⁻¹²^ τ^−1/2^. This result establishes the proposed VCSEL as a compact, scalable, and high-coherence laser source suitable for next-generation atomic clocks and quantum sensing platforms. The work paves the way for the widespread adoption of chip-scale frequency reference technologies in precision timing, navigation, and quantum metrology, offering a compelling balance of performance, integration, and manufacturability.

## Methods

### Optical simulations

We first calculate the reflectance spectroscopy and the local standing wave profile using the transfer matrix method^16^. For a multiple-layer film under normal illumination, it doesn’t matter what the polarization is, i.e., s-polarization or p-polarization. With incident electrical field at $z={z}_{0}$

1 $$E\left({z}_{0}\right)={E}_{r}{e}^{{ik}{z}_{0}}+{E}_{l}{e}^{-{ik}{z}_{0}}$$

and magnetic field

2 $$H\left({z}_{0}\right)=\frac{1}{{Z}_{c}}{E}_{r}{e}^{{ik}{z}_{0}}-\frac{1}{{Z}_{c}}{E}_{l}{e}^{-{ik}{z}_{0}}$$

the output electromagnetic field at $z={z}_{j}$ is derived as

3 $${\left[\begin{array}{c}E\\ H\end{array}\right]}_{z={z}_{j}}=\mathop{\prod }\limits_{j=1}^{m}\left[\begin{array}{cc}\cos {\omega }_{j} & \frac{i}{{h}_{j}}\sin {\omega }_{j}\\ i{n}_{j}\sin {\omega }_{j} & \cos {\omega }_{j}\end{array}\right]{\left[\begin{array}{c}E\\ H\end{array}\right]}_{z={z}_{0}}$$

where ${\omega }_{j}=k{n}_{j}{d}_{j}$, ${d}_{j}={z}_{j}-{z}_{j-1}$, ${h}_{j}={n}_{j}$. Here, the subscript r (l) denotes the light propagates along the +z ( − z) direction, and ${d}_{j}$ is the thickness of the *j*th layer, ${n}_{j}$ is the refractive index of the *j*th layer, ${Z}_{c}$ is the wave impedance. A single layer’s transfer matrix is defined as

4 $${M}_{j}=\left[\begin{array}{cc}\cos {\omega }_{j} & \frac{i}{{h}_{j}}\sin {\omega }_{j}\\ i{\eta }_{j}\sin {\omega }_{j} & \cos {\omega }_{j}\end{array}\right]$$

The Eq. (2) can be rewritten as

5 $${\left[\begin{array}{c}E\\ H\end{array}\right]}_{z={z}_{j}}={M\left[\begin{array}{c}E\\ H\end{array}\right]}_{z={z}_{0}}$$

6 $$M={M}_{m}\cdot{M}_{m-1}\cdots {M}_{1}=\left[\begin{array}{cc}{t}_{11} & {t}_{12}\\ {t}_{21} & {t}_{22}\end{array}\right]$$

The reflective coefficient is described as

7 $$r=\frac{{n}_{a}{t}_{11}-{n}_{s}{t}_{22}+{n}_{a}{n}_{s}{t}_{12}-{t}_{21}}{{n}_{a}{t}_{11}+{n}_{s}{t}_{22}+{n}_{a}{n}_{s}{t}_{12}+{t}_{21}}$$

where ${n}_{a}$ is the refractive index of the outer space, and ${n}_{s}$ is the refractive index of incident region. Moreover, the reflectivity *R* is given by

8 $$R={\left|r\right|}^{2}$$

The cold cavity linewidth can be obtained by the Lorentz fitting of the reflectance spectra.

To acquire the transverse modes behaviors, we calculate the radial intensity distributions of LP_01_, LP_11_, and LP_21_ transverse modes using the effective refractive index method for optical fiber model. The effective refractive index of the VCSEL cavity can be approximated by the intensity-weighted average refractive index of the standing wave field, similar to the step-index waveguide model used in optical fibers:

9 $${n}_{{eff}}=\frac{\int n\left(z\right)* {E}^{2}\left(z\right){dz}}{\int {E}^{2}\left(z\right){dz}}$$

where $n\left(z\right)$ is the material refractive index profile and represents the electric field distribution along the vertical $\left(z\right)$ direction. Assume that the wave vector in the core layer is ${k}_{{in}}={n}_{1}{k}_{0}$, and the wave vector in the cladding layer is ${k}_{{out}}={n}_{2}{k}_{0}$, where ${k}_{0}$ is the wave vector in vacuum, and the propagation constant in the core is ${\beta }_{i}={k}_{{in}}\sin \varphi$, where *φ* is the angle of incidence. To analyze the mode characteristics of the VCSEL equivalent model, the concepts of transverse normalized propagation constant and normalized frequency are introduced. The transverse normalized propagation constants of the core are^36^:

10 $${\beta }_{{ni}}^{2}=\left({k}_{0}^{2}{n}_{1}^{2}-{\beta }_{i}^{2}\right){a}^{2}$$

The transverse normalized propagation constants of the cladding are:

11 $${\beta }_{{no}}^{2}=\left({\beta }_{i}^{2}-{k}_{0}^{2}{n}_{2}^{2}\right){a}^{2}$$

12 $${\beta }_{{ni}}^{2}+{\beta }_{{no}}^{2}={V}^{2}$$

The normalized frequency is *V*:

13 $${V}^{2}={k}_{0}^{2}\left({n}_{1}^{2}-{n}_{2}^{2}\right){a}^{2}$$

Where *a* is the oxidation aperture, ${n}_{1}$ is the refractive index of the core layer, and ${n}_{2}$ is the refractive index of the cladding layer. For a waveguide with a constant longitudinal refractive index, the electric field vectors $E(x,y)$ and magnetic field vectors $H(x,y)$ in its cross section are called mode fields. Light propagates along a waveguide, so it can be assumed that:

14 $$E\left(r,t\right)=E\left(r,\,{{\varnothing }}\right)\exp \left[i\left(\omega t-{\beta }_{i}z\right)\right]$$

15 $$H\left(r,t\right)=H\left(r,\,{{\varnothing }}\right)\exp \left[i\left(\omega t-{\beta }_{i}z\right)\right]$$

Maxwell’s curl equation can be expressed in cylindrical coordinates as:

16 $$i\omega \varepsilon {E}_{r}=i\beta {H}_{{{\varnothing }}}+\frac{1}{r}\frac{\partial }{\partial \varphi }{H}_{{{\varnothing }}}$$

17 $$i\omega \varepsilon {E}_{{{\varnothing }}}=-i\beta {H}_{r}-\frac{\partial }{\partial r}{H}_{z}$$

18 $$i\omega \mu {E}_{r}=-\frac{1}{r}\frac{\partial }{\partial {{\varnothing }}}{H}_{r}+\frac{1}{r}\frac{\partial }{\partial r}\left({rH}{{\varnothing }}\right)$$

According to Eqs. (15), (16), (17) above, we can solve ${E}_{r}$, ${E}_{\varphi }$, ${H}_{r}$, ${H}_{\varphi }$:

19 $${E}_{r}=\frac{-i{\beta }_{i}}{{\omega }^{2}\mu \varepsilon -{\beta }_{i}^{2}}\left(\frac{\partial }{\partial r}{E}_{z}+\frac{\omega \mu }{{\beta }_{i}}\frac{\partial }{r\partial {{\varnothing }}}{H}_{z}\right)$$

20 $${E}_{\varphi }=\frac{-i{\beta }_{i}}{{\omega }^{2}\mu \varepsilon -{\beta }_{i}^{2}}\left(\frac{\partial }{r\partial {{\varnothing }}}{E}_{z}-\frac{\omega \mu }{{\beta }_{i}}\frac{\partial }{\partial r}{H}_{z}\right)$$

21 $${H}_{r}=\frac{-i{\beta }_{i}}{{\omega }^{2}\mu \varepsilon -{\beta }_{i}^{2}}\left(\frac{\partial }{\partial r}{H}_{z}-\frac{\omega \mu }{{\beta }_{i}}\frac{\partial }{r\partial {{\varnothing }}}{E}_{z}\right)$$

22 $${H}_{\varphi }=\frac{-i{\beta }_{i}}{{\omega }^{2}\mu \varepsilon -{\beta }_{i}^{2}}\left(\frac{\partial }{r\partial {{\varnothing }}}{H}_{z}+\frac{\omega \mu }{{\beta }_{i}}\frac{\partial }{\partial r}{E}_{z}\right)$$

Where *μ* is permeability, *ε* is permittivity, *ω* is frequency. According to the assumption, the Helmholtz wave equation becomes:

23 $$\left[\frac{{\partial }^{2}}{{\partial r}^{2}}+\frac{1}{r}\frac{\partial }{\partial r}+\frac{1}{{r}^{2}}\frac{{\partial }^{2}}{\partial {{{\varnothing }}}^{2}}+\left({k}^{2}-{\beta }_{i}^{2}\right)\right]\left[\begin{array}{c}{E}_{z}\\ {H}_{z}\end{array}\right]=0$$

Thus, we obtained the two-dimensional distribution form of the electric field:

24 $${e}_{z}\left(r\right)=\left\{\begin{array}{c}{C}_{1}{J}_{m}\left(\frac{{\beta }_{{ni}}}{a}r\right)r < a\\ {C}_{2}{K}_{m}\left(\frac{{\beta }_{{no}}}{a}r\right)r > a\end{array}\right.$$

Where *m* is the mode order, *J* is the first kind of Bessel function, *K* is the second kind of Bessel function, ${C}_{1}$ and ${C}_{2}$ are undetermined coefficients.

To acquire the stable emission at high temperatures, we calculate the changing of gain peak wavelength, and cavity mode with the temperature using the self-heating (non-isothermal) models of PICS3D software. This involves several key steps. Firstly, we import the thermal conductivity (κ) for each material. Secondly, we introduce Joule heat as the heat source, and the first type of thermal boundary is specified as the thermal contact type. Defining the lattice temperature at the electrode contact points is equivalent to establishing this initial thermal boundary. Thirdly, the setup of heat flow is implemented. Finally, the temperature profile is obtained by solving the heat flow equation. The temperature distribution satisfies the following basic thermal equation:

25 $${C}_{P}\rho \frac{\partial T}{\partial t}=-\nabla \cdot {J}_{h}+H$$

where ${C}_{P}$ is the specific heat, *ρ* is the density of the material, and *H* is the heat source.

### Epitaxial growth

The epitaxial layers were grown using a MOCVD system on 6-inch Si-doped GaAs substrates. The active region comprises three pairs of 6 nm In₀.₁₃₅Ga₀.₈₆₅As quantum wells, each separated by 8 nm Al₀.₂₅Ga₀.₇₅As barrier layers. The oxidation layer was composed of 30 nm Al_0.98_Ga_0.02_As and was located at the first node of standing wave field above the quantum well. The passive cavity of about 4.5λ optical thickness is placed in the first DBR pair adjacent to the active region. The top mirror comprises 23 pairs of p-doped Al₀.₁₆Ga₀.₈₄As/Al₀.₉₂Ga₀.₀₈As quarter-wave DBRs, while the bottom mirror includes 36 pairs of n-doped DBRs with the same composition. To reduce series resistance, intermediate layers with linearly graded compositions were inserted at each interface between high and low refractive index layers in both mirrors.

### Device fabrication

The VCSELs were fabricated using a standard process flow at foundries. First, a ring-shaped Ti/Pt/Au p-type ohmic contact was deposited onto the p⁺ layer surrounding the emission aperture. The p-type DBR layers were then etched using chlorine-based reactive ion etching (RIE) or inductively coupled plasma (ICP-RIE) to expose the high-Al-content oxidation layer. A selective wet oxidation process was carried out at 400 °C in a N₂/H₂O ambient to form oxide apertures with a diameter of approximately 3.5 μm. To achieve electrical isolation, a SiO_2_ passivation layer was deposited via plasma-enhanced chemical vapor deposition (PECVD). A thick Au anode was subsequently electroplated onto the top contact region. The wafer substrate was mechanically thinned to ~200 μm by grinding and polishing. Finally, an Au/Ge/Ni n-type Ohmic contact was evaporated onto the backside of the substrate to complete the device. During fabrication, surface gratings were defined with a 28 nm etch depth, 880 nm period, and a 60% duty cycle.

### Laser linewidth derivation from frequency noise PSD

The intrinsic Lorentzian linewidth of the VCSEL was determined from its frequency noise power spectral density (PSD). The PSD, *S*_ν_(*f*), characterizes the frequency fluctuations of the laser as a function of Fourier frequency *f*. In the high-frequency region where the spectrum becomes flat (white noise floor), the Lorentzian linewidth Δ*ν*_L_ given by:

$$\Delta {v}_{L}=\pi {S}_{v}\left(f\right)$$

where *S*_ν_(*f*) is expressed in Hz^2^/Hz.

The spectral lineshape *S*_E_(*ν*) can be formally related to the frequency noise PSD by:

26 $${S}_{E}\left(v\right)={E}_{0}^{2}{\int }_{0}^{\infty }\cos \left[2\pi \left(v-{v}_{0}\right)\tau \right]\times \left[-4{\int }_{0}^{\infty }{S}_{v}\left(f\right)\frac{{\sin }^{2}\left(\pi f\tau \right)}{{f}^{2}}{\sin }^{2}\left(\pi f\tau \right){df}\right]d\tau$$

where the central optical frequency is denoted as ν₀, and the amplitude of the optical field *E*₀ is assumed constant under the negligible amplitude noise approximation^29^. In practice, the Lorentzian linewidth is extracted directly from the flat region of the PSD using the first equation.

### Measurements

The cross-sectional sample was prepared using a dual-beam focused-ion-beam (FIB) system (ZEISS Crossbeam 540). A TEM system (FEI Talos F200X G2) was used to obtain the TEM images of the device. The L-I-V-PCE characteristics and far-field angles of the VCSELs were measured using a three-station manual probe station system (MPS200-UD). The OPSR of the VCSEL was determined using a polarization-resolved measurement system. The spectral properties were measured with an optical spectrum analyzer (YOKOGAWA AQ6370E). The frequency noise of the VCSELs was verified by an automated cross-correlation homodyne laser noise measurement system (OEwaves OE4000). A Cs atomic clock testbed was employed to measure the frequency stability of the VCSEL. This setup is a closed-loop system where the VCSEL illuminates a Cesium vapor cell. A photodiode (PD) detects the transmitted light to generate an error signal. This signal is used by a microcontroller and a DAC to stabilize the VCSEL’s frequency. A TCXO (Temperature-Compensated Crystal Oscillator) serves as the local oscillator, whose frequency is locked to a 4.6 GHz microwave signal from a VCO (Voltage-Controlled Oscillator) via a PLL (Phase-Locked Loop). The microwave signal, in turn, excites the Cs atoms. This entire feedback loop ensures a stable 10 MHz clock output, which is used to characterize the VCSEL’s stability.

## Supplementary information

SUPPLEMENTAL MATERIAL for 1-MHz Linewidth VCSEL Enabled by Monolithically Integrated Passive Cavity for High-Stability Chip-Scale Atomic Clocks

## Data Availability

The data that support the findings of this study are available from the corresponding authors upon reasonable request.

## Code availability

The code that supports the findings of this study is available from the corresponding authors upon reasonable request.
