# Supplementary material for: 1-MHz linewidth VCSEL enabled by monolithically integrated passive cavity for high-stability chip-scale atomic clocks
Source: Light Sci Appl. 2026 Jan 29;15:94. doi: 10.1038/s41377-026-02192-x (PMC12852856; doi:10.1038/s41377-026-02192-x)
Supplement: Supplementary file 1 — SUPPLEMENTAL MATERIAL for 1-MHz Linewidth VCSEL Enabled by Monolithically Integrated Passive Cavity for High-Stability Chip-Scale Atomic Clocks [file 41377_2026_2192_MOESM1_ESM.pdf]

Supplementary information for

1-MHz Linewidth VCSEL Enabled by Monolithically Integrated Passive Cavity for High-Stability Chip-Scale Atomic Clocks

Zhiting Tang[1], Chuanlin Li[1], Xuhao Zhang[1], Wuyang Ren[1], Kai Shen[1], Chuang Li[1], Qingsong Bai[2], Jin Li[3], Aobo Ren[1*], Hao Wang[4*], Xiaorong Luo[5,6], Hongxing Xu[7], Jiang Wu[1,6,7*]

[1]Institute of Fundamental and Frontier Sciences, University of Electronic Science and Technology of China, Chengdu 611731, China

[2]Chengdu Spaceon Electronics Corporation Ltd., Chengdu 610036, China.

[3]School of Instrumentation and Optoelectronic Engineering, Beihang University, Beijing 100191, China

[4]Division of Electrical Engineering, Department of Engineering, University of Cambridge, Cambridge CB3 0FA, UK

[5]College of Microelectronics, Chengdu University of Information Technology, Chengdu, China

[6]State Key Laboratory of Electronic Thin Films and Integrated Devices, University of Electronic Science and Technology of China, Chengdu 611731, China.

[7]Mozi Laboratory, Zhengzhou 450001, China.

Email: aobo.ren@uestc.edu.cn ; hw447@cam.ac.uk ; jiangwu@uestc.edu.cn

This PDF file includes SUPPLEMENTARY FIGURES (Figs. S1-S10)

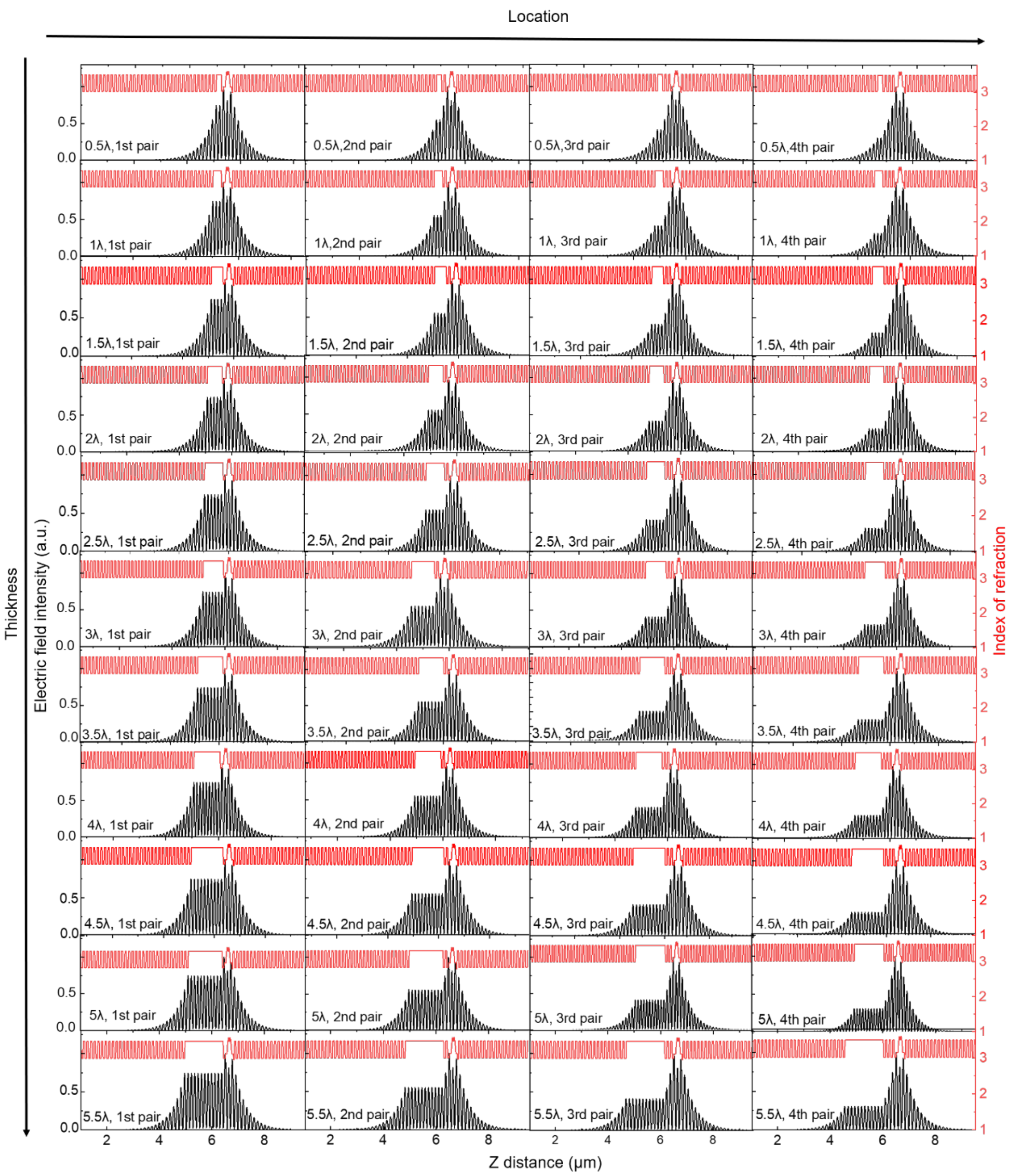

Figure S1. Calculated distribution of the refractive index and normalized electric field of VCSELs. This panel illustrates the position of the embedded passive section (P_m, where m = 1, 2, 3, …) within the distributed Bragg reflectors (DBRs), along with the corresponding passive cavity length (L_p). The electric field intensity profile reveals how the optical mode overlaps with the passive section, informing optimal placement for enhanced photon confinement and reduced optical losses.

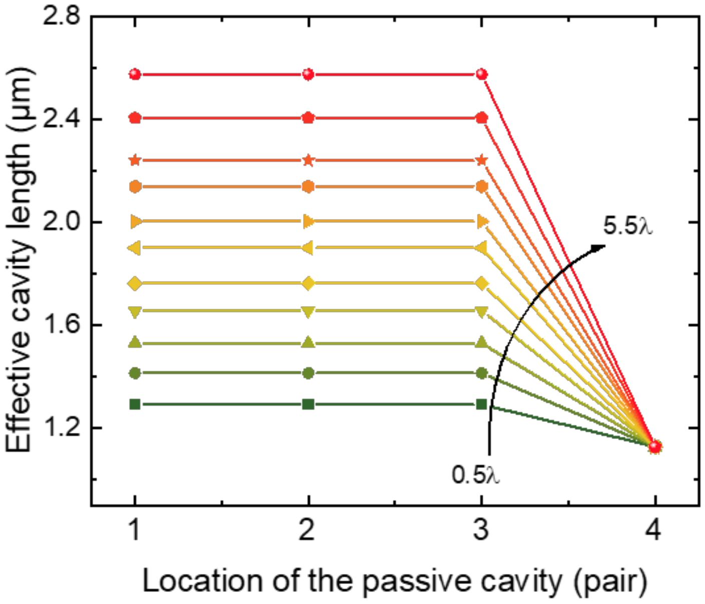

Figure S2. Calculated L_{eff} distribution in passive-cavity embedded VCSELs. The variation of L_{eff} as a function of the L_p and the P_m within the DBRs.

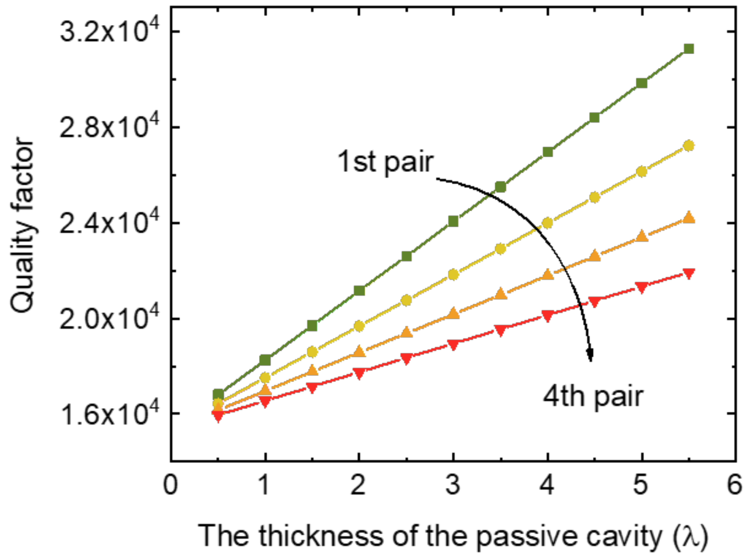

Figure S3. Calculated *Q*-factors in passive-cavity embedded VCSELs. The variation of *Q-factors* as a function of the L_p and the P_m within the DBRs. The *Q-factors* is determined from the full width at half maximum (Δλ) of the resonant dip in the simulated reflection spectrum, where $Q = \lambda/\Delta\lambda$.

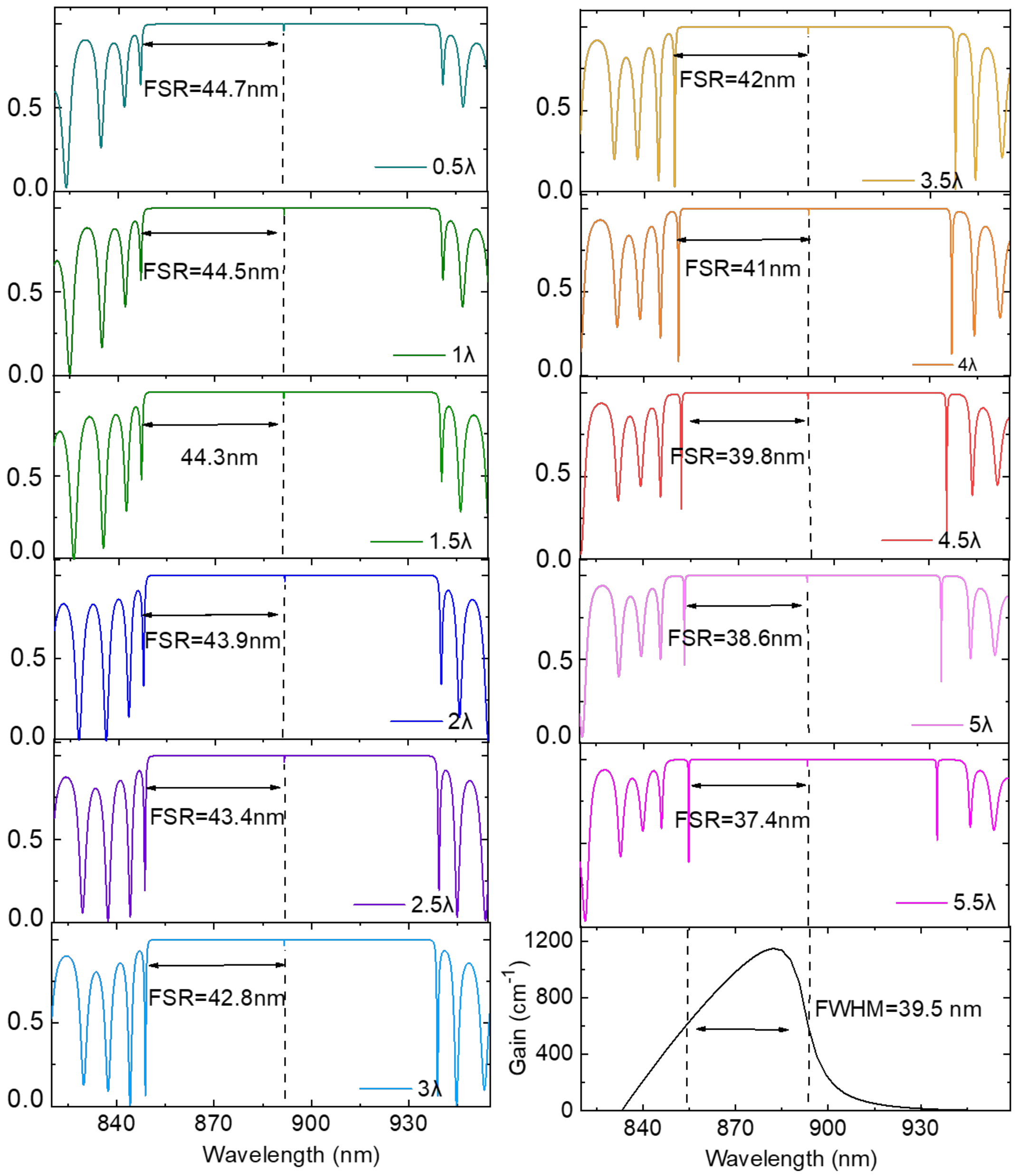

Figure S4. Reflectance spectra and PL spectrum aligned to the central FP dip of passive-cavity embedded VCSELs. Simulated reflectance spectra for different passive cavity lengths, illustrating the appearance and evolution of Fabry–Pérot (FP) longitudinal modes. The variation in layer thickness modulates mode spacing and reflectance bandwidth, providing insights into optimal cavity design for single-mode operation. The longitudinal cavity mode appears near 891.6 nm, while the PL spectrum peaks at 882.1 nm with a full width at half maximum (FWHM) of 39.5 nm—significantly broader than the mode spacing for a cavity length of 5λ or greater.

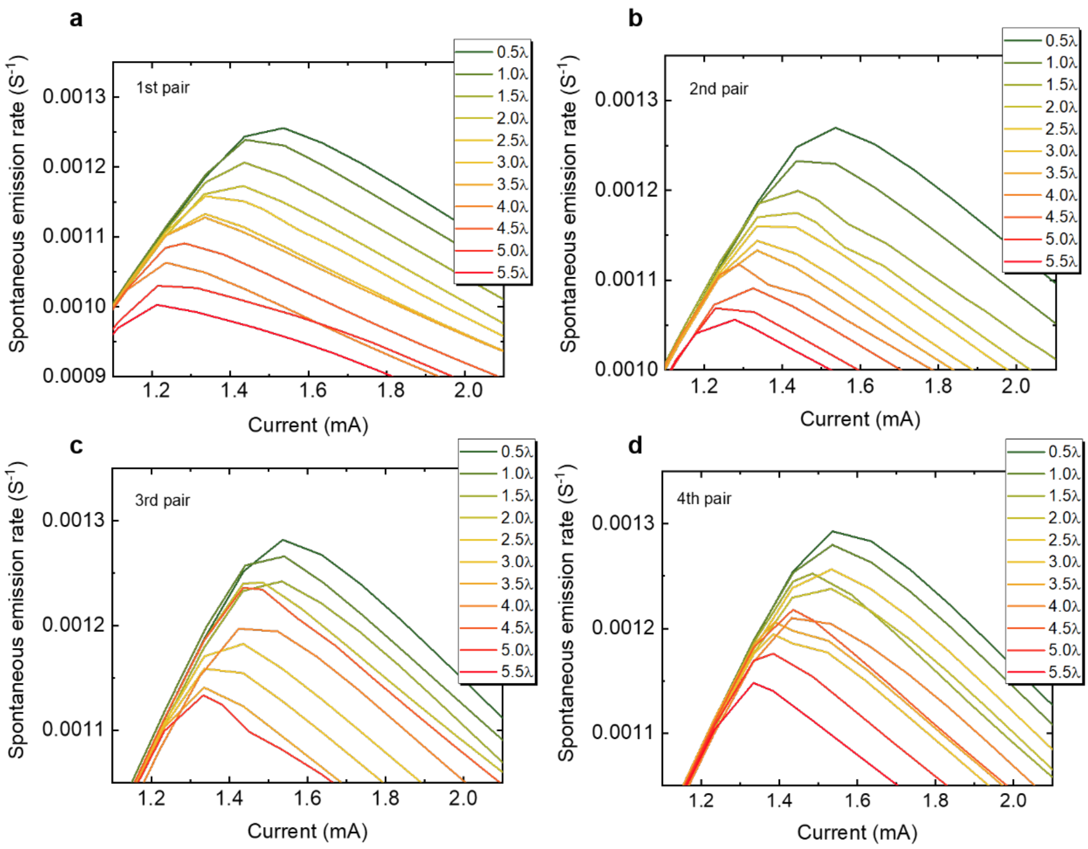

Figure S5. Spontaneous emission rates across various cavity configurations. Calculated spontaneous emission rates for different L_p and the P_m within the DBRs. The results confirm that optimized passive-cavity configurations effectively reduce spontaneous emission, contributing to linewidth narrowing and enhanced coherence.

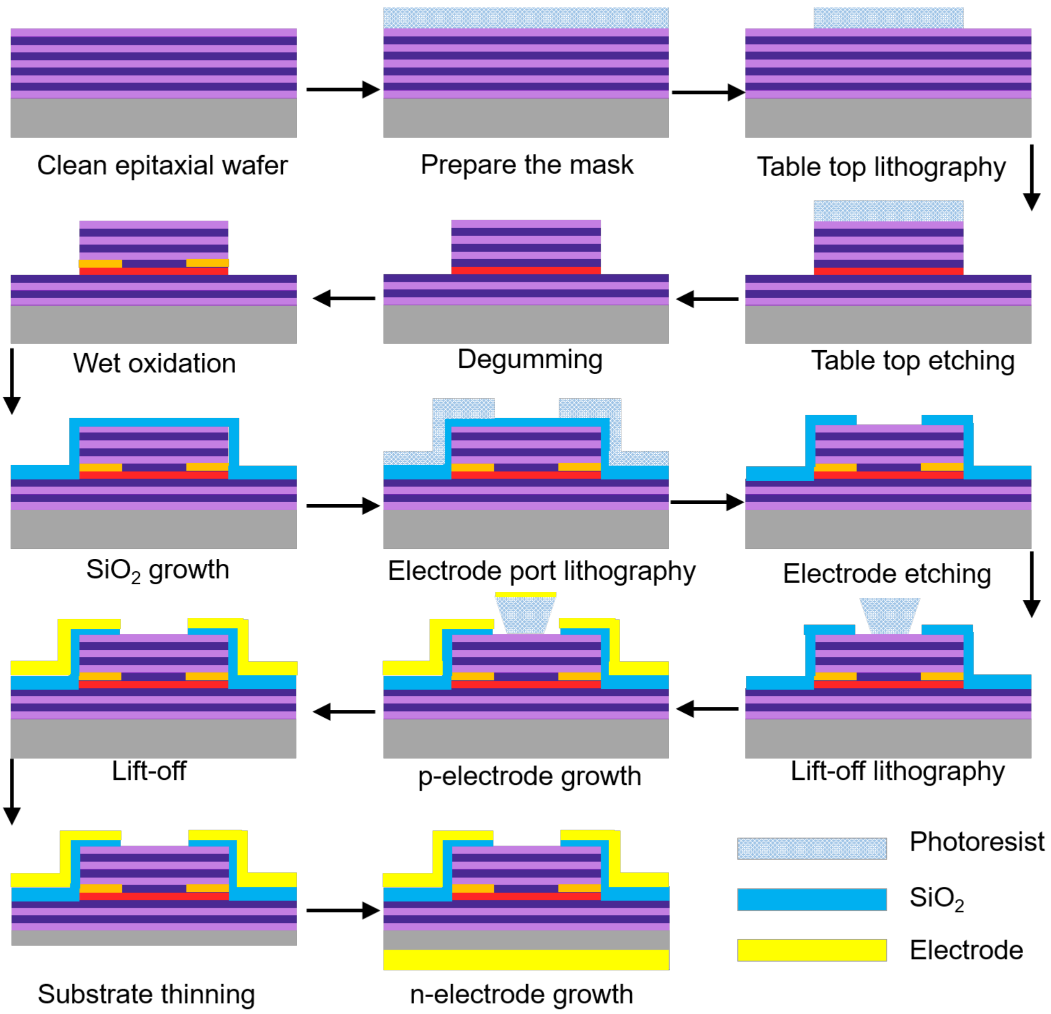

Figure S6. Standard III–V wafer processing flow for VCSEL fabrication. First, a ring-shaped Ti/Pt/Au p-type ohmic contact was deposited onto the p^+ layer surrounding the emission aperture. The p-type DBR layers were then etched using chlorine-based reactive ion etching (RIE) or inductively coupled plasma RIE (ICP-RIE) to expose the high-aluminum-content oxidation layer. A selective wet oxidation process was performed at 400 °C in an N_2/H_2O ambient to form oxide apertures approximately 3.5 μm in diameter. For electrical isolation, a SiO_2 passivation layer was deposited using plasma-enhanced chemical vapor deposition (PECVD). A thick gold anode was subsequently electroplated onto the top contact region. The wafer substrate was mechanically thinned to ~200 μm by grinding and polishing. Finally, an Au/Ge/Ni n-type ohmic contact was evaporated onto the backside of the substrate to complete the device.

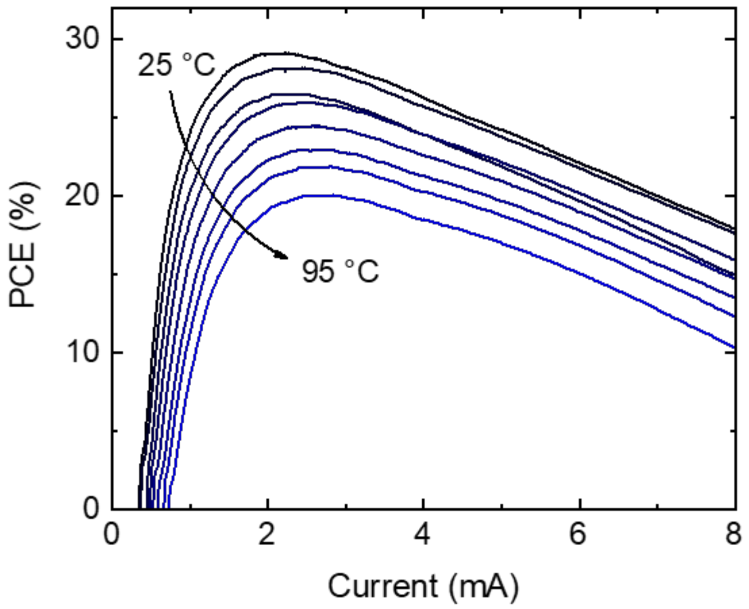

Figure S7. Power conversion efficiency (PCE) characteristics of the proposed VCSEL. Measured PCE of the VCSEL under different ambient temperatures ranging from 25 °C to 95 °C, showing thermal effects on device efficiency.

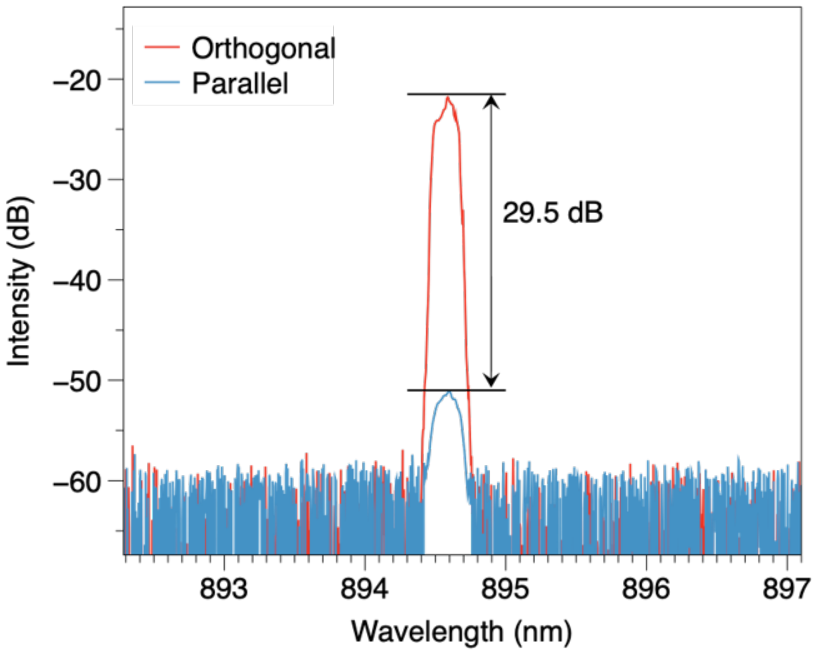

Figure S8. Orthogonal polarization suppression ratio (OPSR) measurement. Polarization-resolved emission spectra of the VCSEL measured at a bias current of 1.4 mA and an ambient temperature of 75 °C, demonstrating strong suppression of the parallel polarization mode.

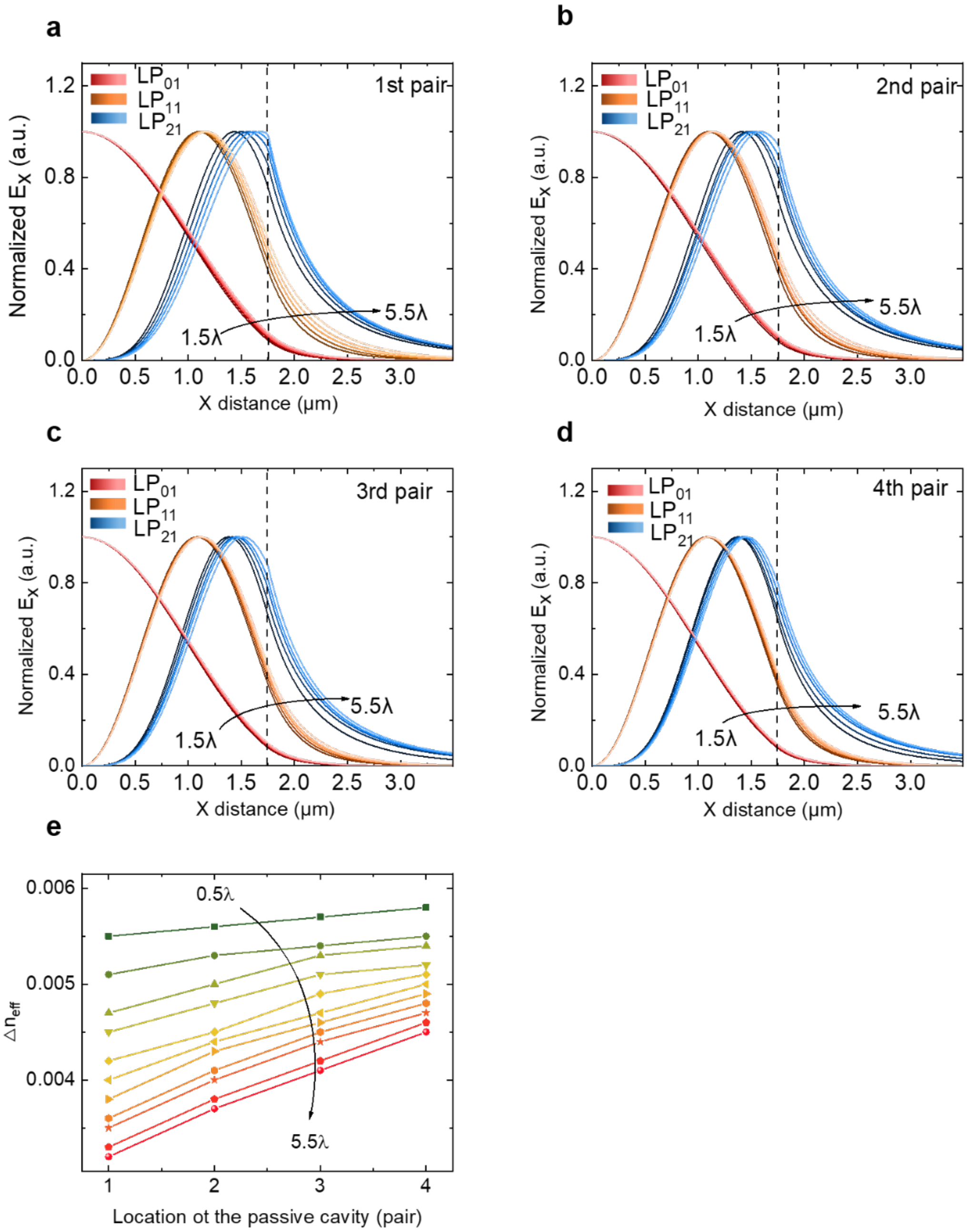

Figure S9. Calculated effective refractive index difference (Δn_{eff}) of passive-cavity embedded VCSELs. Simulated Δn_{eff} values for various passive cavity lengths. The results show that the proposed VCSEL structure exhibits the smallest Δn_{eff} among all configurations considered, enabling optimal suppression of higher-order transverse modes.

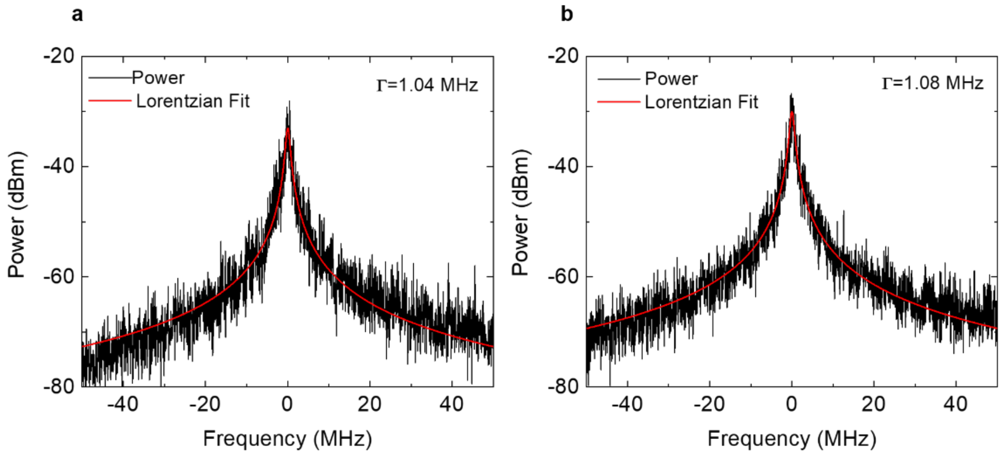

Figure S10. Heterodyne beat note measurement of the proposed 894.6 nm VCSEL. For linewidth characterization, the 894.6-nm VCSEL was driven by an ultra-low-noise isolated voltage source (SRS SIM928) and temperature-stabilized using a TEC controller (Thorlabs TED200C). A narrow-linewidth external-cavity diode laser (Toptica DL pro, <1 MHz free-running linewidth near 895 nm) was tuned to a frequency approximately 1 GHz below the VCSEL emission. The two beams were combined with a 50:50 non-polarizing beamsplitter (Thorlabs BS014), coupled into single-mode fiber (Thorlabs SM800-5.6-125), and detected using a 2-GHz Si fiber-coupled photodetector (Thorlabs DET025AFC, 400-1100 nm). The heterodyne beat signal was analyzed on a Keysight N9030B PXA signal analyzer, with the resolution bandwidth set to 30-100 kHz and trace averaging enabled in order to resolve the ≈1.0-MHz linewidth. A Lorentzian fit to the beat-note spectra yielded VCSEL linewidths of approximately 1.04 MHz at 25 °C (**a**) and 1.08 MHz at 85 °C (**b**).
